# Bibliographical Notices

**Published:** 1849-04

**Authors:** 


### BIBLIOGRAPHICAL NOTICES.

*The Maternal Management of Children in Health and Disease.* By Thomas Bull, M. D., Member of the Royal College of Physicians, Author of "Hints to Mothers for the Management of their Health," &c. &c. From the third London Edition. Philadelphia, Lindsay & Blakiston, 1849.

The above work is especially intended, as its title shows, for the public. It is a treatise on the *maternal* management of the sick and well child. It is intended also, as we are informed in the preface, not "merely for those who are within the reach of good medical and surgical advice, but for those whose lot has been cast in uncivilized places, and where they are totally unable to obtain professional assistance.

After a careful perusal of the work we are satisfied that the publishers have conferred a favour upon the mothers of the country, by placing within their reach a book that cannot fail to be of great assistance to all who have the charge of young children. No one who has much to do with the diseases of early life, or who is consulted by parents as to the hygienic management of children, but must have met with a vast amount of ignorance upon the simplest points connected with these subjects. The physical management of the child devolves almost wholly upon the mother in the great majority of instances, and yet there are few who think of asking the advice of the physician upon this point, or even of taking much serious thought upon the matter, until some dangerous or fatal experience has taught them the necessity of so doing. There can be no doubt, however, that a great deal, probably a large moiety of the sickness and suffering that occur during infancy and childhood, and much of the heavy mortality which takes place during these periods of life, depend upon causes either wholly or in part under the control of human means. Upon the influences acting upon the first few years of existence, depend very often not only the life of the child, but the degree of perfection or imperfection of its present health, and not unfrequently of the health of the individual during long subsequent years. How important, therefore, is it to remedy the defective methods of management which entail such serious and fatal consequences upon the human race. The mode of doing this is by spreading abroad amongst the people a conviction of their existence, and a knowledge of the means of removing them. This important result can be obtained but in two ways;—by the constant teachings of physicians, and by promoting the reading of works devoted to these subjects. There must be some guide by which women may regulate their treatment of children, and we know of none better than some such treatise as the above for that purpose.

No mother could study the above work without acquiring knowledge that would greatly assist her in her duties. It would teach her to avoid many dangers upon which thousands yearly make wreck of all their fondest hopes. It would save her many anxious hours, and her child much suffering and discomfort, for, we repeat,—the health and lives of the young are, to a very great extent indeed, influenced by the manner in which they are fed and clothed, by the in-door and out-door arrangements to which they are subjected, by the judicious exhibition or witholding of what are called domestic remedies, and, lastly, by the manner in which they are cared for and tended, when disease in any of its manifold shapes comes, as come it must, with very rare exceptions, to all members of the human family.

We know of no work upon these subjects, better suited to the mass of the people, than the one before us. It is written in a plain, concise and perspicuous style. It addresses itself to the common sense of the reader, and is evidently the production of a man who has seen much practice, and thought much upon the themes which he discusses.

The work is divided into two parts, the first being devoted to the consideration of the management of children in health, the second to their management in disease. The topics constituting the first part are maternal nursing, wet nurses, artificial feeding, diet from the second year to the eighth, general management (including apartments and servants, sleep, bathing, clothing, air and exercise,) medicines, their use and abuse, vaccination, and lastly, management during teething and hints on the permanent teeth.

The second part includes general remarks on illness, hints for the early detection of disease in the child by the mother, accidents and diseases which may occur at birth, or soon after, accidents of infancy and childhood, (as burns, bruises, wounds, &c.,) diseases of the stomach and bowels of infants, and lastly, the diseases of children, as colds and coughs, constipation, worms, eruptions, &c.

The first part of the work is that which has pleased us most, as it includes what is especially the province of the mother, the care of the child in health, and thereby the *prevention* of disease. We deem it unnecessary, indeed we have not the space to enter into a critical analysis of the work under consideration. Suffice it to say, that we regard it as in every way worthy the notice both of parents and physicians.

---

*Lectures on Venereal and other Diseases arising from Sexual intercourse, delivered in the Summer of* 1847, *at the Hôpital des Midi, Paris.* By M. Ricord. Reported and translated by Victor de Meric, M. D., M. R. C. S. E. Philadelphia, Edward Barrington and G. D. Haswell, 1849.

These valuable lectures were first published in the London Lancet, and the American publishers deserve the thanks of the American medical profession for having collected them in a neat and well printed volume. The reputation of M. Ricord in this department of medicine, is so well appreciated as to guarantee for the work a cordial reception from the members of our profession. M. Victor de Meric, the reporter and translator, has performed his task with great ability. The work is written in a concise and agreeable style, and should be in the library of every medical man.

---

*On the Cryptogamous Origin of Malarious and Epidemic Fevers.* By J. K. Mitchell, A. M., M. D., Professor of Practical Medicine in the Jefferson Medical College of Philadelphia. Lea & Blanchard, 1849. pp. 137. 12mo.

At last, and we cannot forbear from congratulating our professional brethren on the occasion, a public teacher of the practice of medicine has formally discarded the untenable dogma of miasm, so far as this last is regarded, as the product of vegetable or of vegeto-animal decay and decomposition, and as itself the cause of periodical fevers. We say dogma, advisedly, for the propositions which it contained were so little in accordance with the material facts in the case, and so little in harmony with each other, that they could not be regarded in the light of even an ingenious hypothesis, still less a plausible theory. There is not a single condition assumed for the production and evolution of miasm, that is not contradicted by its appearance, we should rather say by the occurrence of fevers implying its appearance in the opinion of the miasmatists, under quite different if not opposite circumstances,—as of soil, temperature, quantity of vegetable or of vegeto-animal remains, &c.

Doctor Mitchell merits our thanks, not merely for refusing his assent to error—and this is a good deal in the search after truth—but, also, because he opens out and carries us along a new road, which, although it may not actually lead to the desired goal, presents, indubitably, much to instruct at the time, and to suggest farther investigations in the same direction.

The professor of the Jefferson Medical College presents his facts and his views on the cryptogamous origin of periodical and other endemic and some epidemic fevers, in six lectures, which were delivered to the graduating class in January 1847, and which, retaining nearly the same shape, are now published in a small volume. He tells us: "Previously I had not put my ideas on the subject of which they treat into so formal a shape, although I had announced for years to each successive class, my impression that, probably, the protophytes might afford a good explanation of the causation of malarious and other diseases of a febrile nature." In his " Introduction and Dedication to the Candidates for Graduation in the Jefferson Medical College, of the Session of 1846—1847," in which he makes this remark, he introduces a letter from Professor J. W. Bailey, dated West Point, March 5, 1845, in answer to one from the author, and in which that gentleman expresses the interest he felt in Dr. Mitchell's letter on the fungous origin of fevers, and his belief that the latter had made out a very strong case. Finally, we read, in the introduction, a letter from a committee appointed by the class of the College, dated December 8th, 1847, in which a formal request is made that the Professor would furnish for publication " his new and original views of the nature and causes of malarious diseases." We are the more particular in repeating these dates, in justice to the author, on the score of whatever merit may attach to originality, or at least priority of promulgation, and connected relation of the prominent facts and details of the subject. Although his views are of more recent publication than those of Dr. Cowdell, on the cryptogamous origin of cholera, yet he has we believe, unquestioned priority over this gentlemen in formally and connectedly teaching to his class this origin, not merely of one disease, but, in addition, of a large class of diseases of a febrile character. That similar opinions had been advanced by "Henle, Müller and others," the author does not attempt to conceal. Berg had described the lamina in follicular stomatitis or thrush, as consisting of an albuminous deposit with a parasitic fungus. The fungiform complication in favus and some other cutaneous diseases had, also, been noticed by different writers. These and other analogous observations are referred to in the "Lectures;" and we might ourselves add hints, found in books, of a similar origin of epidemic fevers. But, the chief point, in investigating questions of discovery, is not so much germinal hints as the fructification of a doctrine, by which it assumes shape and symmetry, and obtains general notice and study, it may be assent and conviction. Examined by this test, we cannot deny that now, for the first time, is the profession made acquainted with a connected and ingeniously sustained theory of the cryptogamous origin of endemic and epidemic fevers, in the six published lectures by Dr. J. K. Mitchell.

In imitation of the advocate who begins to plead the cause of his client, we shall first state the case, by affirming its leading features, and promising to prove their truth subsequently by the adduction of corroborating evidence. In doing so, we shall invert the course pursued by the author himself, and shall give his own conclusions first, because we believe that in this way our readers will be sooner placed in possession of the main outlines of the subject, and be the more disposed, after having seen its extent and importance, to follow with becoming care in the line of requisite details.

"While I was impressed," (writes Dr. Mitchell,) "for the reason so ably stated by Holland with the greater probability of the organic theory, I prefer, for reasons stated by myself, the fungous, to the animalcular hypothesis.

"My preference is founded on the vast number, extraordinary variety, minuteness, diffusion and climatic peculiarities of the fungi.

"The spores of these plants are not only numerous, minute, and indefinitely diffused, but they are so like to animal cells, as to have the power of penetrating into, and germinating upon, the most interior tissues of the human body.

"Introduced into the body through the stomach, or by the skin or lungs, cryptogamous poisons were shown to produce diseases of a febrile character, intermittent, remittent and continued; which were most successfully treated by wine and bark.

"Many cutaneous diseases, such as *favus* and *mentagra*, are proved to be dependent upon cryptogamous vegetations; and even the disease of the mucous membrane, termed aphthæ, arises from the presence of the minute fungi.

"As microscopic investigations become more minute, we discover protophytes in diseases, where, until our own time, their existence was not even suspected, as in the discharges of some kind of dysentery, and in the *sarcina* of pyrosis. We are therefore entitled to believe that discovery will be, on this subject, progressive.

"The detection of the origin of the muscardine of the silk worm, and a great many analagous diseases of insects, fishes and reptiles, and the demonstration of the cryptogamism of these maladies, their contagious character in one species of animals, their transfer to many other species, nay even to vegetables themselves, all concur to render less improbable, the agency of fungi in the causation of diseases of a febrile character.

"A curious citation was subsequently made, of the fungiferous condition during epidemics and epizootics. These moulds, red, white, yellow, gray, or even black, stained garments, utensils and pavements, made the fogs fetid, and caused disagreeable odors and spots, even in the recesses of closets, and the interior of trunks and desks.

These moulds existed, even when the hygrometric state did not give to the air any unusual moisture for their sustentation and propagation. Their germs seemed to have, as have epidemics, an inherent power of extension.

"The singular prevalence of malarious diseases in the autumn, is best explained by supposing them to be produced by the fungi, which grow most commonly at the same season. The season of greatest photophytic activity, is, in every country, the period of the greatest malarious disturbance. The sickly season is, in the rains in Africa, in the very dry season in Majorca and Sardinia, in the rainy season of the insular West Indies, and in the dry season of Demerara and Surinam. Even when the vegetation is peculiarly controlled, as in Egypt by the Nile, and the cryptogami are thus thrown into the season of winter and spring, that season becomes, contrary to rule, the pestilential part of the year.

"Marshes are a safe residence by day, whilst they are often highly dangerous by night. In the most deadly localities of our southern country, and of Africa, the sportsman may tread the mazes of a swamp safely by day, although at every step, he extricates vast quantities of the gases, which lie entangled in mud and vegetable mould. This point, so readily explained by reference to the acknowledged nocturnal growth and power of the fungi, is a complete stumbling-block to the miasmatists.

"The cryptogamous theory well explains the obstruction to the progress of malari offered by a road, a wall, a screen of trees, a veil or a gauze curtain.

"It also accounts for the nice localization of an ague, or yellow fever, or cholera, and the want of power in steady winds to convey malarious diseases into the heart of a city, from the adjacent country.

"It explains also well, the security afforded by artificially drying the air of malarious places, the exemption of cooks and smiths from the sweating sickness, the cause of the danger from mouldy sheets, and of the sternutation from old books and papers.

"On no other theory can we so well account, if account at all, for the phenomena of milzbrand and milk-sickness, the introduction of yellow fever into northern ports, and the wonderful irregularities of the progress of cholera.

"The cryptogamous theory will well explain the peculiar domestication of different diseases in different regions, which have a similar climate; the plague of Egypt, the yellow fever of the Antilles, and the cholera of India. It accounts, too, for their occasional expansion into unaccustomed places, and their retreat back to their original haunts.

"Our hypothesis will also enable us to tell, why malarious sickness is disproportionate to the character of the seasons; why it infests some tropical countries and spares others; why the dry Maremma abounds with fevers, while the wet shores of Brazil and Australia actually luxuriate in healthfulness. The prolonged incubative period, the frequent relapses of intermittents, and the latency of the malarious poisons for months, can only be well explained by adopting the theory of a fungous causation.

"Finally, it explains the cause of the non-recurrence of very potent maladies, better than the chemical theory of Liebig; and shows why the earliest cases of an epidemic are commonly the most fatal."

Let us next inquire into the successive steps by which the author has been led to these conclusions: and first of the composition and diffusion of the fungi. Of the different groups of the cryptogamia, the fungous is the one which Dr. Mitchell believes to be the potent cause of the fevers and other diseases referred to on the present occasion; but he does not entirely deny "the agencies of cognate beings of kindred subdivisions, which are hardly distinguishable from it."

*Proximate and Ultimate elements of Fungi.*—"Of all vegetable substances the fungi are the most highly animalized. Like animals, they disengage carbonic acid and imbibe a quantity of oxygen; nay, some of them extricate hydrogen, and even nitrogen. Their chemical composition also allies them to animal structures. They yield the vegetable products, resin, sugar, gum, fungic acid, and a number of saline compounds; but they also afford the adipocire, albumen, and osmazome of the animal kingdom. The basis of these plants is fungin, a tasteless but highly nutritious substance, white, soft, and doughy. It yields, by nitric acid, nitrogen, hydrocyanic, oxalic, and some other acids, and fatty substances, like wax, tallow, and, in some instances, oil.

*Diffusion and Number of Fungi.*—Of the cryptogamous plants, the *fungi* are distinguished for their *diffusion and number*, for their *poisonous properties, and their peculiar season of growth*, for *the minuteness of their spores*, and for *their love of darkness and tainted soils, and heavy atmospheres.*"

And again:

"A mushroom growth is proverbial in every language. In a single night, under favourable circumstances, leather, or moist vegetable matter, may be completely covered with mould. Of the more minute fungi, some species pass through their whole existence in a few minutes, from the invisible spore to the perfect plant. Lind says, that the first rains in Guinea have been known to make the leather of shoes quite *mouldy* and rotten in forty-eight hours; showing that the plants which disorganize the leather must have drawn their nutrition, even from its heart, in that time, and, by many successive generations, extended themselves over its total surface. Mr. Berkeley describes a *Polyporous squamosus* which, in three weeks, acquired a circumference of seven feet five inches, and a weight of thirty-four pounds. The *Polyporous frondosus* described by John Bapt. Porta, sometimes transcends a weight of twelve pounds in a few days.

"The *Bovista giganteum*, on the authority of Carpenter, the eminent physiologist, has been known to increase in a *single night*, from a mere point to the size of a large gourd, estimated to contain four thousand seven hundred millions of cells; a number which when counted at the rapid rate of 300 per minute, or five per second, would take the whole time of one person, *night and day, for three hundred years.* A square mile contains upwards of 3,000,000 square yards, or 27,000,000 square feet, so that a single *Bovista giganteum* may present, at evening, an almost invisible single cell, and yet place before morning, nearly 1,800 such cells in every square foot of a square mile.

"Notwithstanding the wondrous productions of a *single* individual of one *species*, Fries, the Swedish naturalist, observed not less than *two thousand species*, within the compass of a square *furlong*. The same author tells us, that he has counted above 10,000,000 of sporules in a single individual of the *Reticularia maxima*, so minute as to look like smoke as they rose in the air.

"Webster, when writing of the malignant fever of 1795, informs us that *sound potatoes from market* perished in his cellar, in thirty-six hours; and we know now how they perished. It was a parasitic death.

"In the *Philosophical Transactions*, Lond. (vol. iv. p. 308, Abridg.,) it is stated, that a green *mould* attacked a split melon, and took three hours to sprout, and six to ripen and produce, and let fall new seeds.

"At New York, the pestilential season of 1798, Webster says, that he saw a cotton garment covered with dark gray-colored spots of *mildew in a single night*, and that such events were then and there common."

The family of fungus is, indeed, as Badham terms it, immense: it spreads itself most inconveniently over our dried preserves, makes bread mouldy, destroys, in the form of rust, our grain, rots and fattens upon our fruits, and destroys the careful gleanings of the pains-taking botanist. Nor need we wonder at this, when we learn that its members prey upon each other, or attach themselves to insects, so as to form investments and appendages larger than the animal itself. Then, as regards habitation, the fungi are found in every climate; some families among them infesting "only the steppes of Tartary," and others revelling "solely in the sands of Zahara. *This ubiquity is one of their most peculiar qualities.*"

But why is it then, if the same fungi create disease in Lapland and Senegal, that there is so fatal a difference in the intensity of them at these two places? To this question of the author he replies by saying, that it is not necessary for us to suppose that the same species are every where the cause of malarious fevers; and, moreover, that climate will modify the activity of their properties, so that "*the poisonous cryptogami are rendered yet more poisonous by increased temperature and moisture.*"*

That the sporules of the fungi are absorbable into the circulation, is affirmed by the author; and if we consider the minuteness of these bodies, their volume, being "one-third of that of blood globules, and two-thirds only of that of chyle globules," and the fact of poisons finding their way into the circulation, and be it added of these sporules being detected in the secretions, we can hardly refuse our assent to the assertion.

*Season of Greatest Activity of the Fungi.*—The coincidence between the greatest frequency of malarious diseases (at the end of summer and in autumn) and the exuberant growth of fungi are pointed out. The insalubrity of a place is declared to have the most constant relation to the habits of the living vegetation. It occurs when the phanerogamous or flower-bearing plants have completed their annual task of growth, flowering and fruitage, and when, at the same time, the cryptogamous or non-flowering plants acquire the ascendency at the expense of the other great division. Of one hundred and five species of fungi treated of by M. Roques, "ninety-two are active in autumn and thirty-six in summer."

The generally recognized fact of the comparative exemption from the operation of the causes of periodical fever during the day, and their terrible frequency and intensity during the night, is adduced by Dr. Mitchell as being connected with the active growth of the fungi during the nocturnal period. To the same purport is the contrast between the fatality from sleeping on shore in sickly climates, and the exemption of those who remain on board ship, or return to the vessel before night sets in. "In the extraordinary tendency of fungous vegetables to develope their power only at night, we detect *another analogy between malaria and the fungi.*" A little farther on we are told, that "these anomalous vegetables not only choose for their growth the *season of vegetable repose, but the hours of vegetable* SLEEP."

The electrical relations of fungi to the mist of a marsh, are referred to in connection with their rapid nocturnal growth and power.

* The italicised portions of all our extracts are so in the text.

*Circumstances Modifying the Growth of Fungi.*—Slight changes in their external relations modify greatly the existence and properties of the fungi, and yet their germs resist causes of destruction of the most active nature. Boiling water, many of the acids, and caustic ammonia fail to destroy them, and they sustain the cold of carbonic acid. A weak solution of the albumen of an egg, slightly acidulated, will give rise to various *monilia*, but when it was made alkaline the genus Botrytis appeared. On a neutral or simple solution, no fungi showed themselves.

The spouting of fungi, according to Pereira, occurs from organized and generally decayed or decaying substances, "*not perfected when entirely immersed in water.*" Even minerals support each a peculiar cryptogamous vegetation. Some fungi are confined to particular plants, both above and beneath the surface of the ground, and some, as the entophytes, exist only in the *interior* of living vegetables. Even hard dried wood is not proof against their incursions—witness the dry rot in timber. The same plant is sometimes found to be innocuous when produced during the day, and noxious when of a night growth. So subtle and fugacious is the poison of the cryptogami, that a little daylight or sunshine may totally alter its properties.

Doctor Mitchell nexts points out "the extraordinary association of fungous life with the existence and propagation of great epidemics and intense endemics." In this part of his subject he quotes a number of writers who have recorded the presence of "moulds," of a white, yellow, gray, or even black colour, adherent to the roofs of houses, pavement of cities, to articles of wearing apparel, and domestic utensils of wood; also of "blood rain," a red blue earth brought down with showers, and the rapid decay of fruit, at the same time, with plagues, pestilence and violent fevers. "In pestilential Africa, when the rains and the sickness commence together, the fungiferous powers are fearfully developed."

According to Park and Lind, the first rains stain the clothes, and make even woolens and leather *mouldy*, and rotten in a day or two. The like extraordinary tendency to the production of moulds has been noticed in different parts of the United States, during the period of epidemics of yellow fever; and also in cholera. So, likewise, was it in the *sudor anglicanus*, and in epizootics of cattle. The author argues with much plausibility in favour of a similar connection between fungi and *milk-sickness*, a disease which, in the western regions of our country, has within the last quarter of a century elicited so much conjecture and speculation regarding its origin. He describes its symptoms, and shows that " the animals originally affected are only such as live upon herbage, such as ewes, horses, goats and sheep," on the unbroken soil of a new country, and in the end of summer and in autumn. The poison, whatever it is, acts only *at night*, but it is not particularly modified by temperature or hygrometric state of the atmosphere. It is received into the stomach of cattle as food or beverage. Reasoning on all the circumstances of its operation, Dr. Mitchell is convinced of the virus being *organic*, and of vegetable origin, "and among vegetables we find none, whose habitudes and modes of action so strongly as the fungi, entitle them to the sad distinction of creating this singular malady."

In the next lecture, the author shows the poisonous quality of fungi, and that they produce, when eaten, diseases resembling marsh fevers. Tertian fever has followed the eating of mushroom. The poisonous operation of diseased potatoes, and of ergot, is referred to fungiform growth in the tuber and in the seed, respectively, of the potatoe and the rye. The phenomena of ergotism are shown to resemble often the symptoms of malignant fever and cholera ; as also those from eating spoiled meat to be analagous to typhoid fever.

Periodicity is another effect of the poison of fungi. The author attributes an attack of tertian fever, from which he himself suffered, to the inhalation of the spores of various species of fungus, over which he leaned for several hours a day, watching the changes with a microscope, and measuring the relative size of their spores and nucleoli. He cites the observation of Merat and Lens, that "*preparations of the bark are the best remedy*" for disease produced by fungi.

" More intense poisonings by superadding buboes and *mortification* to other symptoms, bring fungiform diseases into close resemblance to the *plague*. Indeed, when we read of the course and character of most epidemics, and then turn to the history of *cryptoganism*, in its diversified groupings, we cannot fail to be surprised at the many points of resemblance." These are pointed out, while reference is made to different pestilential epidemics and gangrenous fevers, in different periods and places, described by writers.

*Mode of Introduction of the Poison of Fungi.*—Most of the analogies which Dr. Mitchell has described, are of cases in which the poison was introduced into the stomach, and that in large doses too, and hence it has been objected that the poison could not be received into the system in any other way. To this the author replies in some detail, and we shall repeat his language on the occasion.

"That objection seems more specious than sound, when we remember that very small doses of poisons are highly effective when inhaled by the organs of respiration. Thus a very few drops of chloroform will, by inhalation, produce effects on the nervous and vascular systems, more potent than can be created by any dose, however great, thrown into the stomach. A drachm of ether inhaled from a bag, will intoxicate, stupefy, and prodigiously excite him whom ten or even twenty times that quantity would not greatly move by the stomach. So, while it requires not less than thirty grains of arsenic (Christison) to kill an adult, I have known nearly fatal results from the inhalation of less than half a grain of arseniuretted hydrogen. Now it is obvious that, of the small quantity of the respired articles mentioned, a much smaller quantity is absorbed by the pulmonary membrane, and passes into the circulation. Of the few drops of chloroform used, at least nine-tenths must be exhaled by the breath, and thrown away. But when organized substances find their way into the tide of blood, and that too with vital energies capable of reacting on the elements of the sanguine current, it requires but little acquaintance with physiological and pathological phenomena, to induce us to dread the most fearful results. Even when their vital powers are destroyed by mechanical or chemical processes, vegetable poisons act, in the smallest portions, with great violence. How much strychnia, or digitalia, or aconita is requisite for the disturbance of functions, or the arrest of vital action? Certainly much less than we may readily suppose could be inhaled by a sleeper, if such things were suspended in his atmosphere, even with faint diffusion. But the experiment of Prout during the cholera in London, in 1832, if to be relied on, showed a gain in atmospheric specific weight of one sixty-second part; which would give scarcely less than a drachm by weight of some poison suspended in each cubic foot of the atmosphere of London. That quantity of air may be inhaled during common respiration, in fifty inspirations; and, as most persons respire not less than fifteen times a minute, a cubic foot of air may pass through the bronchial tubes in three minutes and a half. How much, then, of such a poison, may be presented to the bronchial surface, in the course of a single night! With how much more force, too, will it act, when it assails the system through that channel! Substances presented to the gastro-intestinal surfaces are mixed up with the various secretions, mucus, saliva, gastric juice, bile, pancreatic liquor, and special exudations from the peculiar glands of each successive section, while aërial poisons, unmixed and unfettered, are applied at once to a surface on which, behind scarcely a shadow of a film, circulates the blood prepared, by the habitual action of the respiratory function, to absorb almost every vapour, and every odor, which may not be too irritating to pass the gates of the *glottis.* It is, perhaps, for this reason, that we have so instinctive a dislike of mouldy smells, and of humid musty places, and unhappily, we discover, that *in the abodes of filth and poverty, where misery dwells, and moulds do most abound,* the great non-contagious epidemics find and destroy the greatest number of victims, because there is the especial domain of fungiferous potency."

Some details follow respecting "the association of fungous growths with the cutaneous and mucous diseases, both of men and animals;" but which, however interesting, and, to many of our readers, new, we have not room to repeat. We may mention here, by the way, that the extent and frequency of the extracts already made, arise from the abundance of specifications and interesting facts on which the main argument rests, and not from any intention or desire to substitute our present notice for the lectures themselves. They are too valuable and too rich, to allow of any substitute, be it analysis or summary, which we of the editorial or critical craft may devise.

Many of the diseases of the lower classes of animals are shown by the author to be obviously dependent on the assaults of the cryptogami, as in the instances of the *muscadine* of the silk worm, and of other growths on fish and water fowls. Chapter xiii. and xiv. of Leviticus, are quoted in farther illustration of this part of the subject.

*Latency and Limitation of the Cause of Periodical Fever.*—Between these phenomena and the often long period that elapses after the ingestion of fungi, before their poisonous effects are produced, and their occasional limitation to very narrow limits, a striking analogy exists, as is shown in the fifth lecture in the volume now before us.

"Authors have admitted that malaria appears to act in many instances as if it could exert no power, except when close to the spot where it originated, whilst in other cases, it seems to be wafted to a great distance from its apparent source. If we suppose the existence of germs susceptible of reproduction, and progressive growth, these seeming contradictions fall at once. The interruption of progress by a road or wall justifies this view of the mode of conveyance, and the many facts which show the narrow limits of the poisonous activity, enforce it strongly. The place, the very spot, where the disease is found, must reproduce the cause of it for itself, and if the conditions of growth are not present, then will the spot be exempt, even if very near to the most poisonous places. Thus may we, and only thus, explain the occurrence of agues, yellow fever, and cholera, on only one side of a house, or one end of a room, or one side of a street, or wall, or road. A wind may indeed waft the spores in small quantity to a distance, but unless there are there the conditions essential to an adequate reproduction, the spores must lie dormant and harmless. For such reproduction, the marsh mist may be one of the most important elements, but that alone will not suffice, since we know that the disease is not proportional to its frequency or intensity. Other and very local conditions seem to exercise a peculiar power. Thus a new house is known to resist disease better than an old one, and a residence protected by an annual cultivation, immediately around it, is more safe than one which is encircled by lawns in grass. During some unusually sickly years, when scarcely an inhabitant of the skirts of the city escaped marsh fever, the wind set, often for a long period, directly from the infected regions into the heart of the city. In perhaps half a minute from the time when the south-western air left the meadows and pestilential borders of the town, it had crept into every chamber of the place; yet physicians here well know that no disease of a malarious character invaded these chambers, which were, most of them, left open during every night of the sultry autumn.

Writers entitled to credit and authority, by position and professional character, assert, that a gauze veil, or a gauze screen in a window, adds much to the security of the wearer or the occupant of a chamber, in even the most unsound places. We can scarcely see how any gas or vapour, simple or compound, could be arrested by such a defence; but it is easy to suppose the detention of organized and comparatively bulky bodies electrical and glutinous, or moist.

The exemption from fevers—the alleged product of a miasmatic atmosphere—by keeping the house dry and warm and avoiding the damp air of night and early morning, is referred to, with the additional remark: "There is no other poison, save that of the fungi, so far as we know, which is thus disarmed by dryness and heat. In any view of the case, the fact is inexplicable, unless we suppose an organic cause, to which the absence of humidity is antagonistic."

The danger of sleeping on damp sheets only occurs, we are told, when they have acquired a *mouldy* smell. The sternutation and temporary coryza from which the scholar often suffers when searching among old books and manuscripts, is explained by the existence and diffusion, at the time, "of numerous organic spores which have grown into power to torment, among the dampness and darkness of the leafy envelopes."

*Propagation of Yellow Fever.*—The mystery attached to the propagation of yellow fever by fomites, and its importation by ships, as recorded by so many writers, some of whom are quoted on the present occasion, may, the author thinks, be elucidated by the supposition "that a tropical fungus, carried off in dark, damp, animalized holds of ships, or in the offensive clothes of sick or dead seamen, may be introduced into the summer clime of unaccustomed places, and there, as it came from, may go to, the shore, and be sometimes reproductive."

"Through this theory of ours, we can easily see why the disease may be imported, why it is imported rarely, and why it makes so slow a progress from the spot to which originally brought. It will, also, explain its non-contagious character, and even its occasional but rare visit to a village or hamlet. It may also account for its apparently spontaneous appearance in such places as Charleston, Savannah, and New Orleans, in which the winter may not be severe enough to kill the germs, but yet may so affect them as to make their reaction difficult or partial.

"It is only thus that we can comprehend how a *perfectly healthy crew* may bring with them, in the closed hold of their ship, the germs of disease, which, after their dismissal, may pestilentially affect the 'stevedores' who discharge her, or only the labourers *who disturb her ballast.*

We can thus, too, explain the *usual pause* between the first set of cases caught by visitors to, or labourers on board the ship, and the attack upon the inhabitants of the vicinity. This curious interval, noticed by almost every writer, occupies about ten to fifteen days, whilst the period of incubation, after exposure to a known source of infection is only five days. (Vaché.)

"This interval is only to be explained by the supposition that germs of some kind, have gained a footing on shore, and have germinated and grown more numerous. *It is the crop in the hold which produces the first set of cases. It is the crop on the land which causes the second.*

"It is only through the action of some organic cause, that we can explain the tenacity of the attachment of yellow fever to certain ships, and these, too, among the cleanest and best aired vessels in the British service. The Sybille had three several epidemic attacks between the 23d of June, 1829, and the middle of April, 1830. Two of these occurred while at sea. In the West India service, certain ships have usually an outbreak on going into even a healthy harbour."

*Cholera.*—The puzzling, because apparently erratic march of cholera, its sudden irruption and as sudden disappearance, at one time going sluggishly, at another flying on the wings of the wind, often seeming to take its origin in low, damp, illy ventilated houses on land, and sometimes meeting, as it were, a ship in the midst of the broad Atlantic, are graphically sketched by the author, who shows that contagion cannot explain these anomalies and contradictions; and that, although the animalcular hypothesis promises at first an elucidation, yet on further inquiry it too fails us. We are met by "the apparent absurdity of supposing that animalculæ of tropical origin could exist and procreate in a Russian winter. The want of proof that animalculæ are poisonous, or that they fulfil the conditions for such a theory, has been already stated."

"But if we assume for cholera a fungous origin, all difficulties vanish; and, as in the case of yellow fever, an easy explanation may be given of every apparent incongruity. We have only to suppose, what is known to happen in other cases, that the fungi, on which cholera is assumed to depend, acquire at times, as do the germs of some contagious diseases, an unusual power of reproduction and diffusion, a greater potency of expansion. Such germs may be carried by men, and goods, and ships, or may make a slower progress by their own unaided activity, or be scattered by the winds, to regerminate, wherever special conditions are found. Thus can we see why the poison prefers the route of streams, or infests the damp parts of cities; and why classes living in clean apartments in dry districts, suffer so little.

"We can see why women escape better than men, why both cholera and yellow fever, by the natural tendency of the vegetable cause to the organs of generation, almost always cause miscarriage of pregnant women, and why, when a city or country is unhealthy, the fungiferous causes of death, by over-stimulating the organs of re-production, usually make a compensation by the births, for the unusual mortality.

"Can we not thus explain the appearance of contagion, where there is no contagion, and the absence of contagion while there is an obvious conveyance of the epidemic poison from place to place?

"We are no longer surprised to learn that cholera advanced regularly from the tent nearest to the water, to the others successively, until it reached the end of the lines; nor do we feel astonished that it was, in another case, confined to the tent nearest the tank, or to the flank company, or the brigade on the left or right of the army. We now see why ninety men detached from a large corps, and attacked on the first night of absence, on the borders of a lake, were, without damage to the corps, promiscuously mingled again with it, after being brought back, totally disabled, to the original encampment. We can understand now, how, in the Odinka Hospital, whose salubrity was previously proved by the absence of cholera during an epidemic at St. Petersburgh, its eight hundred inmates continued in their usual health, despite the introduction from without of five hundred cases of cholera. We can see how a corps, in its march through an irregularly infected country, may acquire and lose the cholera several times; how a healthy corps may enter a sickly army, *en route*, and not suffer from the prevailing malady. The diffusion, the limitation, the leaving the infection behind, or the carrying it forward, all admit of an easy explanation, if we assume the hypothesis that germs or spores, created exteriorly to the body, are the *semina morbi*, and that they are liable to the usual accidents by which seeds are conveyed or lost, or favoured or repressed."

The author, continuing his work of explanation, feels himself bound to give a reason for the extraordinary exemption of Brazil, New Holland, and the Polynesian Islands, from malarious diseases. With the apparent elements present, these countries, it is alleged, escape the inflictions that might be expected. Of all the hypotheses hitherto offered, none afford, he thinks, a shadow of explanation. Admitting the fungous theory, we can solve the difficulty, and are prepared to expect the exceptions. "Under such a view, we are not astonished at finding Brazil healthy and Africa pestilential; for their obvious, much more, their minute vegetation, is so dissimilar as to render a difference in their invisible phytology highly probable."

These considerations lead the author to an enquiry, why the febrile diseases of various countries differ so much. After some explanatory remarks he asks: "Is it then a matter of special wonder, if a fungus with one set of properties should germinate in India, another in Egypt, and a third in Cuba ?"

His practical inference, in regard to quarantine, is in accordance with the belief entertained by many intelligent physicians of another creed of yellow fever and cholera origin, viz: "that the detention of the sick, is, for yellow fever and cholera unnecessary; while the importance of detaining and purifying cargoes, and soiled baggage, becomes apparently more imperative."

The growth of various diseases of a common character before the irruption of a great pestilence, is, in the opinion of the author, explicable by the supposition of an exotic *fungus* having its powers developed by the fungiform tendency at home. "Similar principles seem to govern the movements of diseases now generally acknowledged to proceed from germs," such as small-pox, measles, scarlatina, and hooping cough. The occasional outbreaks of these diseases "seem to depend rather on germinal power than extrinsic enforcement, and remind one of the locusts, which, though every year present in small numbers, appear by myriads at periods of from seven to seventeen years."

The sixth and last lecture opens with some apposite remarks on the explanatory character of the cryptogamous theory, reconciling contrasts, such as between the low, wet, marshy land of the African "Coast," and the high, dry, free from perceptible moisture, and chiefly pasture grounds of the Maremna of Tuscany, and the Campagna of the Roman States. The volcanic character of the soil of the Italian region just mentioned is alluded to, and its adaptation to fungous growth described, to which the ordure of cattle, the grasses of all pasturages, and heavy dews, also, contribute a share. The anomalies and contradictions arising out of a want of obvious connection in time between the alleged cause, miasm and the like, and its effects, fever, &c., are emphatically dwelt on by the author, who thinks that "the occurrence of severe malarious diseases in barren places, on rocky heights, and sandy plains, shows that we may more rationally attribute the diseases of febrile seasons, rather to the spur given to the general vegetation, which is also communicated to the *cryptogamia* than to a decomposition, which remains without proof, and which, when obviously most active, fails to excite disease."

The alternation, in different years, of health and disease in the same spot, is, according to the present theory, explicable on the supposition of the migratory character and destruction of their own reproductive powers of the cryptogami.

Dr. Mitchell indulges in a short and instructive digression, by combatting with much force Liebig's chemical explanation of contagious disease being " produced by the action of a species of ferment peculiar to it, upon as peculiar a matter contained in the solids or the fluids of the body; by which means said matter is consumed, and thus is a reproduction of the disease prevented by the want of due material upon which the morbid action may be founded." The author then proceeds to make an argument for the non-recurrence of this class of diseases as producible by the leaving in the system the *exuviæ* of germs.

A more plausible explanation than the preceding is offered, of the greater fatality of the first cases of an epidemic than of those which follow them. Toxication by an atmospherically conveyed fungous poison, like that produced by substances of vegetable origin, as from alcohol and opium, is most violent in those unaccustomed to its operation, and like them becomes less potent by habit, that is by repetition. We like much the remarks which follow this explanation, and which we reproduce here for the benefit of our professional brethren.

"For this reason, medical men, at the commencement of a violent epidemic, are driven too often from a treatment founded on proper principles, into a loose and dangerous empiricism. For the same reason, are they disposed, at a later period of the attack, to rely upon means of cure obviously inert, or improper, because, the lessened mortality smiles an approval. Let any one found his treatment from the first, upon a proper knowledge of the pathology, and a decent regard to prominent symptoms, and he will succeed in the end, not only much better, but also much more satisfactorily to himself, than those who lower themselves to the level of mere empirics. The deaths are at first owing, not to the greater potency of the cause, but to the keener susceptibility of the recipient of the disease. While it increases the severity of the cases, this susceptibility is not greater for our remedies, and therefore we must necessarily have, at the outset, less success."

Reference had been made before in these lectures, to the occurrence of fevers in dry soils almost bare of vegetation, and where, of course, no ordinary decomposition can be received as adequate cause of the sickness and mortality among the inhabitants of such regions. " But," adds the author, " there is a teeming vegetation beneath, and almost at the surface of such places, to which alone can we attribute their diseases."

He asks : " may not the healthful power of the plough be mainly attributed to its destruction of fungous growths of this and of other kinds." " The plough is the especial enemy of the fungi, which either beneath the surface, as truffles, or upon it, as mushrooms, are obviously lessened or extirpated by the constant disturbance of an active tillage."

We have now reached the conclusion of the arguments advanced by Dr. Mitchell, in support of the cryptogamous origin of endemic and epidemic fevers. It has been our endeavour to exhibit them in the connection and order of sequence, as we find them in the lectures, without any interruption by doubts or critical commentary on our part. We are desirous that, in this way, our readers should enjoy a portion, at least, of the pleasure which we ourselves have experienced from a perusal of the volume before us. They will, we are sure, join us in opinion, that the views of the Professor of Jefferson Medical College are exceedingly plausible and ingenious ; and that, in addition to the direct knowledge which they impart respecting the etiology and phenomena of fevers, they are full of valuable suggestions and varied inferences, which promise to lead to renewed investigation aud farther discoveries, both in the direction now opened, and in collateral lines.

That the theory is complete in all its parts, the author does not pretend to affirm. It may be objected, for example, that while the range of cryptogamous productions is almost ubiquitary, that of the fevers supposed to result from them is happily limited. " Almost every mineral, however poisonous, supports," as we learn from Dr. Mitchell, " a peculiar cryptogamous vegetation. Thus we have *hydrocrocis arsenici* in solutions of arsenic, *hyd. barytica* in solutions of baryta. Fungi grow in ink, in water, indeed, in every thing ; and naturalists are yet in doubt whether these seemingly diverse things owe their difference to soil, water, and temperature, or to different germs, each capable of growing only in its restricted field." We would ask, also, whether in the most marked instances of fungiform growth, there are not obvious preexistent extremes or abnormal conditions of atmosphere, which of themselves, might figure as plausibly as the fungi themselves, in the etiology of the fevers which follow. Admitting that if a plant of this class may be noxious as produced at night, and hurtless as developed by day, and that "the fungi are active almost exclusively at night," we cannot lose sight of the great meteoric differences as in temperature, moisture and electricity, between the nights and days—especially great in the season of the most aggravated febrile diseases and of fungiform development. The same remark applies to the coincidence between these growths and the prevalence of periodical fevers in autumn; so that the suspicion naturally obtrudes itself on our minds, that the organic products developed under these circumstances of autumnal and nocturnal periods, and the appearance of these fevers, are epiphenomena,—concurrent effects of a common cause. The tufa or volcanic earth, furnishes a nidus for the growth of fine mushrooms, as related in Lecture vi., p. 120. But this tufa in Calabria, whence it is brought to Naples for the purpose, gives out neither fungi nor fever in the former country; and it is only when placed in "shaded excavations, or in natural caves, or in cellars," that it produces such "vast quantities of the best mushrooms." Bring the inhabitant of Calabria down to Naples, and subject him to similar exposures, and we are afraid that fever would be as readily developed in him as mushrooms in the tufa, even although the man and the earth were placed in different and remote caves and cellars. So, also, of moulds and mildew, and other fungous productions, seen in seasons of epidemics: they are evidences and effects, rather than causes of a pestilential atmosphere, which, be it said, manifests itself by other sensible and significant appearances. The rapid decomposition of animal and vegetable matters, are an effect of the febriferous atmosphere, but not causes of fever: this same atmosphere may give rise to fungi at the same time. Fungi most flourish and abound under circumstances of vegetable and animal decay, in states of atmosphere and vitality, avowedly fitted to encourage this decay. One of the peculiar characters of the fungi, or mushroom tribe, consists in their habitation, which is always upon dead or decaying organized matter; and when they appear upon living bodies, they are generally the indications of a state of previous disease dependent on perverted nutrition of the tissues. In this light, rather than as actual cause, must we regard the presence of cryptogami in certain cutaneous diseases. Some observers, cognisant of these and analogous phenomena have gone so far as to doubt the development of fungi from distinct germs, and to suggest that they are generated, as we believe them to brought into activity and growth, by the processes which are antecedent to their manifestation. In support of this speculation may be adduced the fact that, particular species can, with certainty, be increased by exposing a certain mixture of organic and inorganic matter to atmospheric changes, as the process adopted by gardeners for rearing the edible mushroom; and that particular species of parasitic fungi are confined to particular leaves.*

Tertian fever, we are told by Dr. Mitchell, has been produced by eating mushrooms. The like effect has ensued after eating fish, certain fruits, and, also, after cold bathing and the irritation of the urethra by the introduction of a catheter. Some stress is laid by the author on the intermittent types of diseases caused by ergot, and grain affected by fungous deterioration. But this feature is present in other varieties of poisoning, as, for instance, in that from arsenic. We may remark here, by the way, that the violent effects, including gangrenism, so commonly produced by the ingestion of fungous substances, do not exhibit a picture of the common occurrences in periodical fevers, even though we were to include the worst form—the congestive or pernicious. In this part of his argument, the author, it seems to us, proves too much.

As we cannot doubt that the attention of medical observers will now be directed to the subject, we shall in due time learn how far there is a correspondence in given districts or regions, described with minute topographical accuracy, between the abundance and spread of cryptogamous, or more particularly fungous vegetation and periodical fevers. First, however, we must ascertain whether the spores of the fungi are really poisonous, as we know the entire plant to be; and next, whether the spores, numerous and minute though they be, are so thoroughly and extensively diffused through the atmosphere as to be the ready poison admitted into the system through the respiratory, mucous, and cutaneous membranes.

We had intended to conclude our remarks on the general subject of the etiology of periodical fevers, by references to the first lecture

* Carpenter's Principles of General and Comparative Physiology, p. 61.

in Dr. Mitchell's volume, on the various theories hitherto promulgated on this occasion, and to interweave some sketches of opinions and facts, adduced by Italian and especially Roman writers of the present age, who have been led to differ from Lancisis' hypothesis of certain poisonous emanations from stagnant water and marshes being the cause of periodical fevers; but we find that we have already exceeded the limits we had proposed to ourselves, and we must desist for the present. We might, also, have been induced to make some observations on certain points of medical geography introduced into Dr. Mitchell's lectures; as, for example, on his contrast between the pathological conditions of the inhabitants of Western Africa and Brazil, under what he believes to be the same meteorological conditions. These last we think we could show, are much more divergent from one standard than is supposed by the ingenious and investigating lecturer; and that where there is an approximation in climatic features, there is not wanting a certain degree of community of character in the diseases of the two portions of the Eastern and Western continents. Brazil does not enjoy the exemption from malarious diseases given to it in the volume before us, p. 111–12; and the picture of its climate, as drawn by the traveller Walsh, p. 23–4, is in colours too roseate by far. Dr. Sigaud, in his valuable work* on the Climate and Diseases of Brazil, gives descriptions of medical topography as respects stagnant water and marshes, &c., and of periodical fevers in the maritine provinces of that country, the exact counterpart of which is presented to our observation in North America and in the old world. Not only in the adjoining country, but also in Rio de Janeiro itself, are intermittent fevers rife; and, still more, these diseases manifest, especially of late years, a strong tendency to complications, running into the congested or pernicious fevers. So aggravated are their symptoms, and their readiness to become remittent is so marked, that they have been compared to the remittent fevers of Angola, Algeria and Mozambique. In the provinces of Bahia and Matto Groso, pernicious intermittents are found both in the towns and in the country. The remedy now most relied on by the Brazilian physicians, is that which is most used in the United States and southern Europe, viz.: sulphate of quinine in large doses.

* Du Climate et des Maladies du Bresil, &c.

*Report of the Pennsylvania Hospital for the Insane, for the year* 1848. By THOMAS S. KIRKBRIDE, M. D., Physician to the Institution. Philadelphia, 1849.

*Annual Report of the Officers of the New Jersey State Lunatic Asylum at Trenton, December,* 1848.

Although we have little space to offer for the proper notice of publications of the kind above presented, we would be glad to have the opportunity of adding at least half a dozen others, from more distant institutions, and similar in stamp and character to the excellent reports before us. Such documents are always welcome, were it only for the practical instruction they convey upon a vitally important subject; but we regard them with still greater interest, because, in the picture they exhibit of increasingly useful and prosperous activity at work in each noble charity that sends them forth, they do honour to the people by which those charities are fostered, while they prominently mark a striking and yet growing change of public sentiment in relation to a question which is of the highest moment to humanity at large.

Trite as it may seem, we cannot help repeating the remark, most forcibly suggested by each new advent of such reports as those of Dr. Kirkbride and Dr. Buttolph, that in no branch of science or philanthropy is the intellectual and moral advancement of the times more admirably manifest than in the prevailing attention to the rational study of insanity, and in the ever onward march of the moral, the humanizing treatment of this terrible disease.

The strange discovery, first asserted by Pinel, has at last begun to work its way triumphantly, in spite of selfish fears and prejudices, into the better senses of mankind, that the victim of derangement is neither a beast nor a slave, but a veritable human being, whose claims upon our aid and sympathy are so much the stronger because he is unable to enforce them.

We are also beginning to suspect that the christian law of kindness which teaches man to love his neighbour as himself, is not less bland and potent in its influence upon the withered and distracted heart of the poor patient, than it ought to be upon the feelings and conduct of his once dreaded guardian. It is now generally admitted that under the promptings of the simple but sublime philosophy—the unerring policy of the golden rule—results have become matters of every-day occurrence in the history of insanity, which, but a few years since, would have proved scarcely less startling, even to the well informed, than were the miracles of old. Thus do every year's accumulated observations under the improved management of the present day, demonstrate the wisdom of the application to the care of the comparatively helpless occupants of the lodge or day-room, of the same broad principles of humanity and justice that are supposed to regulate the dealings between man and man in a responsible community. Thus too it is equally well shown that though, in the words of Dr. Kirkbride, "much as has been done to ameliorate the condition of the insane within the last twenty years, there can scarcely be a greater error than to suppose that no further advance is necessary." We have the ruling principle spread out before us in all its beauty and completeness, and we have seen enough of its actual operation to be convinced of its entire sufficiency; but too many of the thousand details of its administration are yet beyond the grasp of practical experience.

The "right course," says Dr. K. has only just been entered. Still in the reports which furnish our excuse for these remarks, ample reason may be found for the belief that both the doctor and his Trenton compeers are at least as far beyond the threshhold of reform as the most forward of their fellows in the work; and that with their ability, zeal, and the means at their command, they are no less likely to maintain their high position.

The Pennsylvania Hospital Report informs us that at the outset of the year 1848, there were remaining in the house 188 patients; admitted 215; discharged and died 203; remaining at close of year, 200. Of the total number during 1848 there were cured 120, much improved 23, improved 24, stationary 19, died 17 (12 male and 5 female,) making in all 203.

In conclusion to these summaries we may add, that Dr. K. presents us with his usual full and elaborate variety of tabular statistics to which we would refer our readers for a mass of valuable information.

Passing over a gratifying account of recent and contemplated improvements of the house and grounds, together with many excellent hints and observations in regard to mental treatment and the means and appliances thereto, and finally some useful suggestions on steam warming and artificial ventilation, we meet with an unusually interesting feature of this year's report. This is a review of the history of the insanity department of the Pennsylvania Hospital from the commencment of its operations, ninety-seven years ago. The insane hospital was first opened in this city in 1752, and continued to receive patients, 4367 in number, until January 1841, when the splendid place at Blockley was thrown open. Since then 1391, including those transferred from the town, have been received; so that 5647 inmates have been admitted in the course of ninety-seven years. The statement of "receipts and expenditures" presents a most flourishing condition of affairs, which will we confidently trust enable them hereafter to maintain an enlarged and liberal system of mental treatment, without the fear of hampered means or the necessity of imposing on the funds now needed for the improvement and support of the general hospital in town.

The New Jersey manifesto makes its first appearance in a well filled pamphlet handsomely embellished with two engravings, one a beautiful perspective view of the buildings and adjacent grounds, and the other a neatly executed plan of the first story of the same imposing structure. From the medical report we ascertain that the house was opened May 15th, 1848, and gave admission during the subsequent seven and a half months of the year, to 47 men and 39 women, one man and two women of whom were discharged cured; so that at the close of December, 1848, 46 men and 37 women remained in the wards. No deaths or other serious accidents occurred, and nearly all the patients have more or less improved.

Under the statistical head, the cases are arranged according to age, domestic state and occupations; also according to form of disease (classed as different affections of the sentiments, and ditto of the propensities,) alleged causes, hereditary influence, duration of attack, and residence of patients.

The remainder of the paper is chiefly occupied, in addition to a brief account of the buildings, &c., with some instructive notes on the causes, forms, and treatment of mental disorders, the most favorable period for cure, and the best modes of committing patients to the asylum. Correct and definite information on these topics is of great importance to every intelligent community; and we rejoice to find our distinguished neighbour thus early and efficiently engaged in pressing its diffusion.

In conclusion we heartily concur with Dr. B. in his earnest hope that he will be properly supported in his determination to maintain "*a high standard of keeping.*" With such assistance and encouragement as he has every right and, we trust, reason to expect from the people of the state, we may rest assured, that under his charge the institution may rank, in its management not less than in arrangements and construction, "among the best public asylums in this or any country."

---

*Summary of the Transactions of the College of Physicians of Philadelphia* from September 6th, 1848, to *January* 2d, 1849, inclusive.

The last quarterly number for 1848, contains a large amount of useful matter, in the shape of Reports of committees and discussions among the Fellows of the College.

In the "Reports" is included a very able one on *Theory and Practice of Medicine*, by Dr. B. H. Coates, in which a sketch of the progress of this branch during the preceding year is given, in relation both to domestic and foreign medicine. In the *domestic* department are included notices of several new medical works and papers. In the foreign department is contained a recapitulation of the information and views from different sources in relation to that "wide spreading scourge of the human race," the Asiatic cholera.

The Annual Report of the *Committee on Midwifery* is replete with interest, giving a very just idea of the progress of obstetrical science during the past year.

An obituary notice of the late lamented Dr. H. Neill, vice president of the College, by Dr. J. M. Paul, is also contained in the "*Transactions*," in which the high professional character and eminently christian virtues of the deceased are forcibly drawn by his biographer.

The varied discussions of the College, eminently practical in their character, we hope from time to time to lay before our readers. The number before us fully sustains the high character of the "*Transactions.*"

The following introductory lectures have been received:

1. *An introductory Lecture to a course on Surgery, to the Medical Class of Geneva College.* By Prof. Bryan.

2. *An Introductory Lecture on the Coinciding Tendencies of Medicines.* By Jared P. Kirtland, M. D., Professor of Surgery and Practice of Medicine and Physical Diagnosis, in the Medical Department of the Western Reserve College, Ohio.

3. *Lecture on Obstetrics and the Diseases of Women and Children.* By Gunning S. Bedford, Professor of Obstetrics and the Diseases of Women and Children, in the University of New York. Session of 1848–9.

4. *A Plea for Obstetrics. Introductory Lecture to the Course of Midwifery, in the Medical Department of Pennsylvania College.* By John Wiltbank, M. D.

The first of the above lectures, contains an interesting account of the life and services of Baron Larrey, one of the greatest military Surgeons of which our profession can boast. We thank Prof. Bryan for this well written and agreeable lecture, and commend it to those of our readers who are anxious to learn something of the character of this distinguished surgeon of France.

The second lecture, by Prof. Kirtland, is on the "coinciding tendencies of medicines." While on the one hand we do not like the term "coinciding tendency," on the other we must admit that this important subject has been ably discussed by the lecturer. In explanation of what is meant by the coinciding tendencies of medicines, Prof. R. remarks, that "every agent capable of making an impression, if employed, during the existence of disease, will exert some influence—it will either *counteract* or *coincide.* In the one event, it will either diminish or cure the disease; in the other, augment or modify it, into some anomalous and more malignant form. The result will depend on the contingencies, whether the article is indicated or contra-indicated and skilfully or unskilfully employed." It is absurd and unphilosophical to say that "this or that article of medicine, will do no harm, if it does no good." No medicine ought to be given without some reason for its administration. It is true, in some cases, the difficulties of diagnosis are so great, that physicians are compelled to resort to an experimental plan of treatment; but this does not render it less obligatory on them, to obey the philosophical injunction, of never prescribing for disease, until we have clearly ascertained its nature. In those cases *where this cannot be done*, it would be better simply to prescribe for some predominant or annoying symptom, than resort to that empirical and destructive principle which teaches *that if a medicine does no good, it can do no harm.*

In order to explain his views, Prof. Kirtland refers to the following among the many instances in which the coinciding tendencies of medicines may create factitious disease :

Remedies will coincide 1st, if not adapted to the fulfilment of the indications ; 2d, if not adapted to the grade of the disease ; 3d, if not timed to the stage of disease ; 4th, if disproportioned in power to the amount of disease ; 5th, if employed under a false diagnosis ; 6th, if their use be continued after they have accomplished certain changes in the system ; 7th, if disproportioned in power to the amount of vitality remaining in the system ; 8th, if not suitably qualified.

All these points are properly illustrated by a reference to numerous cases ; but our limits will not permit us to enter more fully into the consideration of these matters.

The third lecture is devoted to the consideration of obstetric medicine. This department of medicine, comprising within its range, " midwifery proper, the accidents of the puerperal state, and the diseases peculiar to females and infancy," claims from medical students special attention, since upon a knowledge of its its principles and practice will depend the health and happiness of the fairest portion of creation. To discharge duties so important, the student should comprehend accurately the mechanism of labour, the management of its accidents, and the treatment, not only of the puerperal state, but of others peculiar to females. It is almost as difficult to *teach* as it is to *learn* the principles of obstetrics ; hence we are not surprised, when Dr. Bedford states that " he never enters upon the performance of duty in this University without a sentiment of apprehension and distrust." The lecture is written in an agreeable style, and enforces with earnestness and good judgment, the necessity of obtaining a thorough knowledge of the principles of obstetric medicine, before venturing upon the responsible duties which its practice involves.

The fourth lecture is also upon the subject of midwifery. The author's "plea for obstetrics," is written in an earnest and persuasive style, and cannot fail to produce a good effect in directing the attention of the student to the study of this important branch of medicine.

